# Assessing correlates of protection in vaccine trials: statistical solutions in the context of high vaccine efficacy

**DOI:** 10.1186/s12874-019-0687-y

**Published:** 2019-03-06

**Authors:** Andrea Callegaro, Fabian Tibaldi

**Affiliations:** GSK Vaccines, Rue de l’Institut 89, Rixensart, Belgium

**Keywords:** Vaccine clinical trial, High vaccine efficacy, Surrogate endpoint, Correlate of protection

## Abstract

**Background:**

The use of correlates of protection (CoPs) in vaccination trials offers significant advantages as useful clinical endpoint substitutes. Vaccines with very high vaccine efficacy (VE) are documented in the literature (VE ≥95%). The rare events (number of infections) observed in the vaccinated groups of these trials posed challenges when applying conventionally-used statistical methods for CoP assessment. In this paper, we describe the nature of these challenges, and propose easy-to-implement and uniquely-tailored statistical solutions for the assessment of CoPs in the specific context of high VE.

**Methods:**

The Prentice criteria and meta-analytic frameworks are standard statistical methods for assessing vaccine CoPs, but can be problematic in high VE cases due to the rare events data available. As a result, lack of fit and the problem of infinite estimates may arise, in the former and latter methods respectively. The use of flexible models within the Prentice framework, and penalized-likelihood methods to solve the issue of infinite estimates can improve the performance of both methods in high VE settings.

**Results:**

We have 1) devised flexible non-linear models to counteract the Prentice framework lack of fit, providing sufficient statistical power to the method, and 2) proposed the use of penalised likelihood approaches to make the meta-analytic framework applicable on randomized subgroups, such as regions. The performance of the proposed methods for high VE cases was evaluated by running simulations.

**Conclusions:**

As vaccines with high efficacy are documented in the literature, there is a need to identify effective statistical solutions to assess CoPs. Our proposed adaptations are straight-forward and improve the performance of conventional statistical methods for high VE data, leading to more reliable CoP assessments in the context of high VE settings.

## Background

Assessing a vaccine’s ability to induce immune responses that can effectively protect from infection and disease is key. The use of clinical endpoints to assess vaccine efficacy (VE) can be burdensome on the development, licensure, duration and effectiveness monitoring of immunisation trials. Replacing the clinical endpoint of a vaccine by an immunological endpoint can positively impact many of these aspects and considerably reduce costs as a result, as well as facilitate ethical procedures. Indeed if measured appropriately, immunological endpoints are biomarkers that can accurately predict VE on a shorter time scale while using significantly fewer participants compared to clinical endpoint assessments, making them an attractive time- and cost-effective option [1].

The terms ‘correlate’ and ‘surrogate’ of protection are common in the literature when referring to immunological endpoints, but are often used inconsistently, including by regulators and other prominent authorities. The first formal definition of surrogacy was introduced by Prentice in 1989, and was complemented with a set of criteria based on the concept of mediation [2]. Several statistical methods for evaluating surrogate endpoints soon followed as part of the causal inference [3–5] and meta-analytic frameworks [6–8], on which Alonso et al. provided a useful description of their relationship [9]. A hierarchical framework was proposed by Qin et al. to shed clarity on the profuse topic of immune correlates, and to assess their validity as substitute endpoints [10]. In their proposal, three levels of association are distinguished: ‘Correlate of Risk’ (CoR) (1), level 1 ‘specific’ surrogate of protection (SoP) (2) and level 2 ‘general’ SoP (3), where levels 1 and 2 reflect whether the analysed data comes from single or multiple trials, respectively. Specifically, a level 1 (specific) SoP is an immunological measurement predictive of VE in the same setting as the trial in which the vaccine was investigated, while a level 2 (general) SoP refers to a surrogate that can predict VE across a range of different populations and settings [10]. Meta-analytic approaches have been proposed to evaluate level 2 SoPs using data collected from multiple trials [6–8].

Within level 1, Qin et al. further subdivide this SoP into a statistical or principal category, according to the method used for their validation. A statistical SoP is an endpoint that satisfies the Prentice criteria [2], while a principal SoP is defined using a causal inference framework [3–5, 10, 11]. The latter aims to address post-randomisation selection bias by estimating what the vaccine responses would have been if the non-vaccinated group of a trial had been immunised. Such endpoints can be used to predict VE once they are validated and approved by a regulatory body.

In this manuscript, SoP endpoints are referred to as correlates of protection (CoPs). Specifically, we address CoP levels 1 and 2, based on Qin et al.’s following definitions of a CoR as an "immunological measurement that correlates with the rate or level of a study end point used to measure VE in a defined population", and a CoP as a "CoR that reliably predicts a vaccine’s level of protective efficacy on the basis of contrasts in the vaccinated and unvaccinated groups’ immunological measurements" [10]. Moreover, we address the concept of CoPs in the context of a continuous, rather than a threshold approach [1].

Although not common, vaccines with very high efficacy (95% or above) are documented in the literature [12–17]. These include the salmonella typhi vi coniugate [12], or the combined measles-mumps-rubella-varicella immunisation [17]. These trials raised the problematic of assessing CoPs in the context of high VE using classical statistical methods. Indeed, a very small number of cases/infections (corresponding to the vaccinated groups) can trigger considerable issues for such statistical models. There is therefore a need to adapt statistical methods for CoP assessment to the context of high efficacy vaccines. To the best of our knowledge, such tailored approaches are lacking in the literature. The aim of this manuscript is to present statistical solutions and to generate adapted methods to assess CoPs based on Prentice criteria and meta-analytic frameworks (by randomized subgroups such as centers and regions) in single trial setting (STS) with high VE.

## Methods

### Statistical methods for assessing CoPs

The Prentice criteria and meta-analytic approach are two classical statistical methods used for assessing vaccine CoPs. The following sections describe both methods, and our specific adaptations as statistical solutions for high VE settings. The results section shows the performance of our proposed adapted models using simulations.

#### The prentice criteria

The following set of notations will be used throughout the manuscript: *T*_*j*_ and *S*_*j*_ are random variables denoting the true binary and the surrogate endpoints for subject *j*=1,...,*n* and *Z*_*j*_ is a binary treatment indicator.

Key concepts, including the hypothesis-testing approach to the validation of substitute endpoints using randomised clinical trial data, were introduced by Prentice [2]. His four criteria for the validation of a surrogate endpoint can be adapted for vaccine trials as follows:

Protection against the targeted disease is significantly related to having received the vaccine, where the corresponding logistic model (Prentice criterion 1) is given by:

$$logit(P(T_{j}=1))=\mu_{T}+\beta Z_{j}. $$

The substitute endpoint is significantly related to the vaccination status (Prentice criterion 2):

$$S_{j}=\mu_{S}+\alpha Z_{j}+\epsilon_{S_{j}}. $$

where *ε* is the zero-mean normally distributed error term.

The substitute endpoint is significantly related to protection against the clinical endpoint (Prentice criterion 3):

$$logit(P(T_{j}=1))=\mu+\gamma S_{j}. $$

The full effect of the vaccine on the frequency of the clinical endpoint is explained by the substitute endpoint, as it lies on the sole causal pathway (Prentice criterion 4).

1 $$ logit(P(T_{j}=1))=\tilde \mu_{T}+\beta_{S} Z_{j}+\gamma_{Z} S_{j}.   $$

Therefore, criterion 4 is met if the null hypothesis H _01_:*γ*_*Z*_=0 is rejected and the null hypothesis H _02_:*β*_*S*_=0 is not rejected.

Although Prentice’s definition and criteria have been the subject of much debate [1, 4, 18], we decided to apply this approach for its simplicity and frequent usage, as well as its close relation to many of the methods proposed later on. These include the proportion of treatment explained [19], the proportion of information gain [20], and the individual-level surrogacy measured by the information theoretic approach [21].

#### The meta-analytic framework

In this paper, we consider the meta-analytic framework in the single trial setting (STS), in which the units are randomized subgroups such as centers or regions. The meta-analytic approach can be represented by a bivariate mixed-effects model as follows:

2 $$ \begin{array}{lcl} S_{ij}&=&\mu_{S}+m_{Si}+\alpha Z_{ij}+a_{i} Z_{ij}+\epsilon_{S_{ij}}\\ logit(T_{ij}=1)&=&\mu_{T}+m_{Ti}+\beta Z_{ij}+b_{i} Z_{ij}, \end{array}  $$

where *μ*_*S*_ and *μ*_*T*_ are fixed intercepts, *α* and *β* the fixed effects of treatment on the endpoints, *m*_*Si*_ and *m*_*Ti*_ the random intercepts, and *a*_*i*_ and *b*_*i*_ the random effects of treatment on the endpoints in subgroup *i* [6]. For simplicity, we assume no random intercepts here (reduced model).

When the full bivariate mixed-effects approach is used to assess surrogacy, computational issues often occur. One simple solution is to use a fixed effect meta-analysis on aggregated data (two-stage approach) [6]. This means performing separate regression of *S* on *Z* and then *T* on *Z* for each of the subgroups and then doing a weighted linear regression of the *T* slope ($\hat \beta _{i}$) on the *S* slope ($\hat {\alpha _{i}}$)

$$\hat\beta_{i}=\lambda_{0}+\lambda\hat{\alpha_{i}}+\epsilon_{i}, $$

with weights given by $w_{i}=1/\hat Var(\hat \beta _{i})$. In this case, the trial level surrogacy is given by the *R*^2^ of the weighted linear regression. More sophisticated regression models can be used, such as the bivariate random effects model [22, 23].

### Statistical solutions for high vaccine efficacy

Statistical methods for the analysis of rare events are extensively described in the literature [24]. VE can be expressed as follows:

$$VE=1-\frac{P(T=1|Z=1)}{P(T=1|Z=0)}, $$

where *P*(*T*=1|*Z*=1) and *P*(*T*=1|*Z*=0) are the probabilities of disease among vaccinated and unvaccinated individuals, respectively. In the context of high VE where a small number of events are observed in the vaccinated group, methods tailored for rare events can be applied in this specific setting. The following sections detail our proposal for statistical solutions that allow reliable CoP assessments of high efficacy vaccines. Both adapted methods are compatible with standard statistical software including R and SAS.

#### Flexible models for prentice criteria framework

The model assessing Prentice criterion 4 includes the surrogate and the treatment as covariates. When the number of events is small, this model can encounter issues due to lack of fit, leading to erroneous conclusions. To solve the problem of lack of fit, flexible link functions [25–27], could be used within Prentice framework. In this paper, we consider the classical logistic models with flexible (non-linear) effect of the surrogate

3 $$ logit(P(T_{j}=1))=\tilde \mu_{T}+\beta_{S} Z_{j}+f(S_{j},\theta)   $$

where *f*(*S*_*j*_,*θ*) is a non-linear function, such as polynomials or smoothing splines. This flexible model is popular for several reasons including: known properties, interpretability of parameters, easy to fit and implemented in many standard softwares.

### The meta-analytic approach using penalised likelihood

The meta-analytic approach can be applied when multiple randomized subgroups are available for analysis. However, when applying this method in a high VE setting, maximum likelihood (ML) subgroup-specific VE estimates may be infinite, causing classical meta-analytic methods that combine subgroup-specific VE to potentially fail. To overcome this issue, we estimated subgroup-specific VE using the penalised likelihood method. Penalisation, which is equivalent to using proper priors on coefficients, solves the problem of infinite coefficient estimates. To achieve this we applied two approaches: the Firth method [28], and the weakly informative prior (WIP) proposed by Gelman et al. [29]. Firth showed that his method is equivalent to the use of Jeffreys’ invariant prior. Gelman et al. on the other hand proposed a WIP distribution (Cauchy prior with scale 2.5), which relies on the assumption that a typical change in an input variable is unlikely to correspond to a change as high as 5 on the logistic scale. As part of a two-step approach, we first independently executed the Firth method and Gelman approach using the logistf and bayesglm R packages respectively [30, 31]. In a second step, we evaluated the performance of both methods as part of a meta-analysis in the context of high VE, by running simulations.

## Results

### Flexible models for the prentice criteria framework

To evaluate the impact of the lack of fit corresponding to Prentice criterion 4, we simulated data using the Dunning regression model [26] in an ideal CoP setting, where the treatment effect is fully explained by the surrogate (full mediation) as follows:

$$P(T_{j}=1|\pi,S_{j})=\pi\frac{e^{\mu+\gamma S_{j}}}{1+e^{\mu+\gamma S_{j}}}. $$

Here, *π* is interpreted as the probability of being exposed to the disease. Irrespective of the interpretation of *π*, this is a valuable, monotone, skewed, flexible and non-linear model to generate the type of data described above.

Simulations were run using the following parameter assumptions: Total sample size *n*=5000, 1:1 randomization, *π*=0.1, *p*_0_=*P*(*T*=1|*Z*=0)=0.05, *μ*_1_=*E*(*S*|*Z*=1)=4.5,4,3.75,3.33, *μ*_0_=*E*(*S*|*Z*=0)=3, *VAR*(*S*|*Z*=1)=*VAR*(*S*|*Z*=0)=0.2, *γ*=*log*(1−0.95), *μ*=8.3. A range of VE values were considered (VE = 0.4, 0.75, 0.85 and 0.95), and 5000 datasets were simulated for each scenario. We fitted Prentice model 4 on the simulated data using classical logit regression shown in Eq. (1), the proposed non-linear model depicted in Eq. (3) with a quadratic term

$$logit(P(T_{j}=1))=\tilde \mu_{T}+\beta_{S} Z_{j}+\gamma_{Z} S_{j}+\gamma_{Z,2} S_{j}^{2}. $$

and the scaled logistic model [26]. Table 1 shows the outcome of these simulations.

**Table 1:** Prentice framework simulation results

logit model 4	$\hat {VE}$	*p*(*S*)<*α*	*p*(*Z*)<*α*	*p*(*S*)<*α* & *p*(*Z*)≥*α*
Linear	0.41	1.00	0.05	0.95
Non-linear	0.41	1.00	0.05	0.95
Scaled logit	0.41	1.00	0.03	0.96
Linear	0.75	1.00	0.10	0.90
Non-linear	0.75	1.00	0.04	0.96
Scaled logit	0.75	1.00	0.04	0.96
Linear	0.86	1.00	0.22	0.78
Non-linear	0.86	1.00	0.05	0.95
Scaled logit	0.86	1.00	0.04	0.96
Linear	0.96	1.00	0.34	0.66
Non-linear	0.96	1.00	0.04	0.96
Scaled logit	0.96	0.99	0.03	0.96

Power (*α*=0.05) to assess Prentice criterion 4 using classical (linear) and flexible (non-linear) model 4 in case of full mediation (data generated using scaled logit model 3). $\hat {VE}$: estimated VE; *p*(*S*)<*α*: power to detect the Surrogate effect; *p*(*Z*)<*α*: type-I error of the treatment effect; *p*(*S*)<*α* & *p*(*Z*)≥*α*: power to meet Prentice criterion 4

Table 1 shows that using a flexible model considerably increases the power to meet Prentice criterion 4 when the VE increases. In fact, the simple linear logistic model does not control the type-I error of the treatment effect (*p*(*Z*)<*α*) when VE is high. This is due to the lack of fit of the linear effect which is absorbed by the treatment effect, thereby considerably reducing the power to meet Prentice criterion 4. We can see that the scaled logistic model is slightly conservative. Standard errors of this model should be computed by bootstrap [27].

### The meta-analytic approach using penalised likelihood

We considered the meta-analytic approach in a single trial setting. The single trial was split into several relatively small randomized subgroups (such as geographical regions or centers), and these small subgroups were used as units for the meta-analysis. For illustration purposes, we analysed a publicly available simulated dataset containing both continuous outcome and surrogate endpoints [21]. This dataset consists of 50 subgroups characterised by a 1:1 randomization and sample size of 20 per subgroup.

**Fig. 1:**
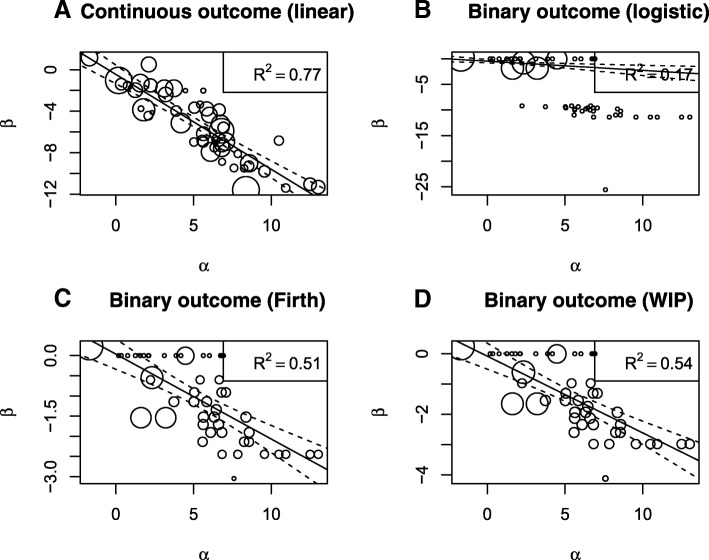
Meta-analytic approach results on Alonso et al.’s dataset (Alonso and Molenberghs 2007). Panels: **a** original data results (continuous outcome); **b** logistic results on the dichotomised outcome; **c** Firth logistic results on the dichotomised outcome; **d** Weakly Informative Prior (WIP) logistic results on the dichotomised outcome

Figure 1a shows the results of the two-stage meta-analytic approach with a continuous outcome. Here, a strong correlation between the treatment effect on the true outcome ($\hat \beta _{i}$) and the treatment effect on the surrogate outcome ($\hat \alpha _{i}$) is observed, with an estimated *R*^2^ of 0.77. When artificially dichotomising the true outcome as *Y*=1 if *T*<−2.87 and *Y*=0 if *T*≥2.87, the resulting VE on this binary outcome is 95%. Figure 1b shows the results on this true binary outcome, where several *β* values fall around -10. These values are extremely high for a logistic regression and they are due to the lack of events in the treatment group, thus generating a small *R*^2^ value (0.17). Figure 1c shows the two-stage meta-analytic approach, where the treatment effect on the binary outcome is estimated using the penalised likelihood approach proposed by Firth [28]. Here, we observe that the problem of infinite estimates is solved, and so the *R*^2^ value is much higher compared to the classical approach. Similar results were obtained using the penalised likelihood approach proposed by Gelman, as shown in Fig. 1d [31]. To better understand the results it is useful to look at summary statistics from the different logistic models by number of events in control and in vaccinated groups. Table 2 shows that when there are no events in the two groups (*n*_*V*_=*n*_*C*_=0) then the estimated effect is zero ($\hat \beta =0$) and the estimated variance is “infinite” for the logistic model while it is relatively small for the penalized methods. When there are no events only in the vaccinated group (*n*_*V*_=0 and *n*_*C*_>0) then the effect and the variance estimated by the standard logistic model are “infinite”, while the penalization of the likelihood prevents infinite estimates and variances. This is the reason why the penalized methods outperform the standard logistic approach in the case of high VE.

**Table 2:** Alonso et al. [21] dataset with dicothomized outcome. Results of logistic, Firth and WIP model by number of events in Control *n*_*C*_ and number of events in Vaccinated group (*n*_*V*_)

*n*_*C*_	*n*_*V*_	Logistic		Firth		WIP	
		$\hat \beta $	$\hat Var(\hat \beta)$	$\hat \beta $	$\hat Var(\hat \beta)$	$\hat \beta $	$\hat Var(\hat \beta)$
0	0	0.00	2.33e+09	0.00	1.15	0.00	1.28
1	0	-9.18	7.85e+06	-0.60	0.79	-0.59	0.65
2	0	-9.59	7.85e+06	-0.91	0.72	-0.90	0.58
3	0	-9.36	2.89e+06	-1.14	0.69	-1.15	0.57
4	0	-9.58	2.89e+06	-1.34	0.68	-1.37	0.58
5	0	-9.78	2.89e+06	-1.52	0.68	-1.59	0.61
6	0	-9.99	2.89e+06	-1.71	0.68	-1.80	0.64
7	0	-10.21	2.89e+06	-1.90	0.69	-2.04	0.69
8	0	-10.98	7.85e+06	-2.13	0.72	-2.33	0.77
9	0	-11.38	7.85e+06	-2.45	0.79	-2.73	0.92
10	0	-25.57	2.33e+09	-3.04	1.15	-3.84	1.91
1	1	0.00	5.60e-01	0.00	0.42	0.00	0.35
3	1	-0.67	4.00e-01	-0.54	0.33	-0.49	0.26
9	2	-1.79	4.30e-01	-1.53	0.35	-1.51	0.30
2	3	0.27	2.80e-01	0.23	0.26	0.21	0.21

To confirm these results, additional data was simulated with a true binary outcome and a continuous surrogate, using the reduced model in Eq. (2) without random intercepts. This dataset consists of 25 subgroups and n =40 participants per subgroup with a 1:1 randomisation. We simulated data using the following parameters: *μ*_*S*_=4.609; *μ*_*T*_=−2.2401; *α*=5.458; *β*=(−1,−2,−4); Var(*a*_*i*_) =10; Var(*b*_*i*_) =4. The correlation between the treatment random effects is $\rho = {Cor}(a_{i}, b_{i})=\sqrt {0.9}$, with an *R*^2^ value of 0.9. The *R*^2^ estimated by different methods as a function of VE is presented in Table 3.

**Table 3:** Meta-analytic simulation results (1000 replications)

Model	*V* *E*	mean (*R*^2^)	median(R^2^)	Std (*R*^2^)	95%ll	95%ul	MSE (*R*^2^)
Logistic	0.75	0.59	0.61	0.16	0.24	0.84	0.12
Firth	0.75	0.72	0.73	0.09	0.54	0.86	0.04
WIP	0.75	0.71	0.72	0.09	0.51	0.85	0.05
Logistic	0.82	0.52	0.54	0.22	0.03	0.85	0.19
Firth	0.82	0.73	0.75	0.09	0.52	0.87	0.04
WIP	0.82	0.71	0.72	0.10	0.48	0.87	0.05
Logistic	0.9	0.46	0.49	0.26	0.01	0.86	0.26
Firth	0.9	0.72	0.74	0.10	0.48	0.88	0.04
WIP	0.9	0.70	0.71	0.11	0.45	0.87	0.05

Estimated R^2^ (mean, median, standard error, 95% confidence intervals and MSE) for different models and values of VEs

Table 3 shows that penalised approaches (Firth and Gelman’s WIP) outperform the standard logistic model in terms of Mean Square Error (MSE), especially in case of high VE where there is a high chance of having subgroups with zero events in the vaccination group. In fact, when the VE is 0.75, 0.82 and 0.95, the average number of subgroups with zero events in vaccination groups are 9, 13 and 20, respectively. Both penalised approaches show very similar results.

## Discussion

Despite recent advances in immunology, we are only beginning to understand how vaccines work best, and how we can improve vaccine design for higher protective efficacy [32]. Although not common, vaccines with a high efficacy, are documented in the literature [12–17, 33]. These include the salmonella typhi vi conjugate [12], or the combined measles-mumps-rubella-varicella immunisation [17]. Rare events data obtained in high VE trials make it challenging for statisticians to apply classical methods used for CoP assessment due to the lack of available information. These include ML estimators, where bias, infinite estimates, multicollinearity and convergence issues can arise and negatively impact Prentice criteria and meta-analytic frameworks commonly used to assess vaccine CoPs, as shown in this paper [24, 26, 27].

To overcome this problem, we evaluated the impact of high VE using two classical statistical approaches: the Prentice framework and the Meta-analytic framework applied on randomized subgroups (e.g. geographical regions). We chose these methods for their common usage in CoP assessments, and their user-friendly characteristics. We performed data simulations with high VE to illustrate the problems and to evaluate the proposed solutions.

By working on the Prentice framework, we show that it is critical to both design and evaluate flexible and adaptable models that are tailored to high VE cases, as the lack of fit of a model leads to substantial loss in power. Accordingly, we propose to analyse data using a logistic model with non-linear surrogate effect. This popular model is flexible, with known properties, easy to fit and implemented in many standard softwares. The number of additional parameters should be small to avoid overfitting. Other models with flexible link functions have also been proposed that can be used within the Prentice framework [26, 27]. Model selection can be done using the Akaike Information Criterion (AIC) approach. Furthermore, adjustments for baseline covariates can play an important role in improving model fit.

Regarding the meta-analytic framework, we demonstrate that penalised likelihood approaches (such as Firth or Gelman’s WIP) outperform the standard logistic model when VE is high, as they solve the problem of infinite estimates. This problem can occur when VE is high where there is a high probability of observing zero cases in certain subgroups of the vaccinated group, as we have also shown. For simplicity, we used a two-stage approach where treatment effects were estimated for each subgroup using a penalised likelihood approach, followed by a (fixed effect) meta-analysis to combine results from different subgroups. Another possibility is to use a mixed model with WIP or Jeffrey priors. For example, it is straightforward to implement the bivariate model, depicted in Eq. (2), with WIP for the covariance matrix of the treatment random-effects using a Bayesian framework (e.g. WinBugs, JAGS or Stan). Additional simulation studies, comparing one and two-stage penalised approaches, would therefore be worth pursuing to help overcome these problematics in the context of high VE.

It is noteworthy that the concept of a vaccine CoP often refers to the establishment of a protective immunogenicity threshold as alluded to earlier, above which disease acquisition is unlikely to happen. However, relating immunological biomarkers to disease risk and therefore VE can also be made possible as part of a continuous approach, without the assumption of a threshold titre. This manuscript addressed this type of (continuous) approach that employs fitted regression models on antibody titres in vaccinated and non-vaccinated individuals to show the statistical association between antibody titres and disease incidence [1, 26, 34, 35].

Although this study was limited by its use of simulated data only, our results suggest that the solutions we propose substantially increase the power of classical statistical approaches for CoP assessment, when dealing with high VE. Furthermore, they are straight-forward and compatible with standard statistical software.

## Conclusions

Following our observation that CoP assessments for high VE vaccines comes with statistical issues using standard methods, we devised flexible non-linear models to counteract the lack of fit in the Prentice framework, and propose penalized likelihood approaches for meta-analysis. These statistical solutions are easy-to-implement adaptations to both conventional methods for application in high VE cases. Such statistical challenges associated with high VE may have so far been overlooked due to their low occurrence, yet high VE cases exist. For binary surrogates it may be interesting to explore how the individual causal association [9] and the surrogate predictive function [36] perform in the setting of high VE. Finally, evaluating the impact of high VE on the Principal stratification approach should be beneficial to the field, towards improving CoP assessments of vaccines [3–5, 10, 11].

## Abbreviations

AIC: Akaike information criterion
CDF: Cumulative distribution function
CI: Confidence interval
CoP: Correlate of protection
CoR: Correlate of risk
LL: Lower limit
ML: Maximum likelihood
MSE: Mean squared error
SE: Standard error
SoP: Surrogate of protection
UL: Upper limit
VE: Vaccine efficacy
WIP: Weakly informative prior
